# Comparison of the Thermal Reaction of Patients after Conserving Procedures and after Mastectomy to the Radiation Dose Obtained during Radiotherapy

**DOI:** 10.3390/ijerph192316085

**Published:** 2022-12-01

**Authors:** Dominika Plaza, Agnieszka Baic, Barbara Lange, Anna Brzęk, Krzysztof Ślosarek, Agata Stanek, Armand Cholewka

**Affiliations:** 1 Radiotherapy Planning Department, Maria Skłodowska-Curie National Research Institute of Oncology Gliwice Branch, Wybrzeze Armii Krajowej Street 15, 44-102 Gliwice, Poland; 2 Faculty of Science and Technology, University of Silesia, 75 Pułku Piechoty 1A, 41-500 Chorzów, Poland; 3 IIIrd Radiotherapy and Chemotherapy Department, Maria Skłodowska-Curie National Research Institute of Oncology Gliwice Branch, Wybrzeze Armii Krajowej Street 15, 44-102 Gliwice, Poland; 4 Department of Physiotherapy, School of Health Sciences, Katowice Medical University of Silesia in Katowice, Poniatowskiego Street 15, 40-055 Katowice, Poland; 5 Chair and Clinical Department of Internal Medicine, Angiology and Physical Medicine, Medical University of Silesia in Katowice, 41-902 Bytom, Poland

**Keywords:** infrared thermography, radiotherapy, isodoses, isotherms, breast cancer

## Abstract

The main aim of the study was to compare the temperature response of the body to the dose received during breast cancer radiotherapy. The control group consisted of 50 healthy volunteers. They underwent one thermographic examination and compared the temperatures between the left and right breasts. The research group consisted of 50 patients. Based on the treatment plan, the area PTV and isodose was marked on the thermograms. Five thermographs were performed in each patient (before radiotherapy and in each week of treatment). A qualitatively similar increase in mean temperature during treatment was observed in both subgroups in the analyzed areas. The highest increase in temperature was obtained in the third week of treatment. Compared with the value before treatment, the increase in the mean temperature in PTV in patients after partial surgery was 0.78 °C, these values are statistically significant *p* = 0.000055. In the case of post-mastectomy patients, 0.8 °C was obtained, these values are statistically significant *p* = 0.00369. In addition, strong correlation was calculated between isodoses read from treatment plans and isotherms obtained from the analysis of thermal images. In post-mastectomy patients for PTV r = 0.77, 30 Gy r = 0.94, 20 Gy r = 0.96, and 10 Gy r = 0.75. For patients after partial surgery for PTV r = 0.74, 30 Gy r = 0.89, 20 Gy r = 0.83, and 10 Gy r = 0.89. Infrared thermography seems to be a useful method of assessing the thermal response of the body to the dose received during radiotherapy of breast cancer and may be a clinically useful method of assessing the early skin response to radiation.

## 1. Introduction

The method choice for treating patients with breast cancer depends on the established clinical stage. The final therapeutic decisions also consider the general condition, the assessed hormonal sensitivity of the tumor, comorbidities, and the patient’s age. Combined techniques, including hormone therapy, immunotherapy, chemotherapy, and radiotherapy, are the standard [1]. Breast cancer radiotherapy is usually performed based on a two-dimensional planning system (2D), using a 5-field technique, in which the chest wall area is irradiated from two tangent fields. The supraclavicular lymph nodes and three levels of the armpit are then irradiated from two fields: the anterior axillary and posterior axillary area, including the third level of the armpit. Field 5, including the parasternal lymph nodes, is rarely used. Currently, radiotherapy is planned in a three-dimensional system based on the CT examination performed [2,3].

This improves control of the irradiated area and protects healthy organs surrounding the target. The planned target volume of Planning Target Volume (PTV) to be irradiated may contain the following:

- A scarred breast or chest wall (depending on the initially performed treatment);
- Armpit and supraclavicular lymph nodes;
- Parasternal lymph nodes on the irradiated side.

After the irradiation area is introduced into the planning system, the medical physicist draws up a treatment plan using the Treatment Planning System. The goal of medical physicist’s work is to create a plan in which the PTV area receives the desired dose while critical organs are the most protected, according to accurate reports. In the case of breast radiotherapy, critical organs are the lungs, heart, liver, spinal canal, and the head of the humerus. Modern irradiation techniques allow the distribution of isodoses to be adapted to the shape of the PTV determined by the doctor. Only after checking and approving the plan can the patient start irradiating the therapeutic apparatus [2,3,4,5,6].

Radiotherapy must be recognized as the administration of energy to the tissue. That is why temperature changes are expected. However, among the side effects seen after radiotherapy as general weakness, decreased activity, vomiting, and changes in the blood (mainly a reduction in the number of white blood cells and platelets), there might also be seen temperature side effects. In the case of breast radiotherapy, skin lesions are the most common. The skin exposed to radiation may turn slightly pink and peel off due to acute radiation dermatitis and ulceration. Acute radiation exposure may lead to a physician’s decision to terminate radiotherapy early, which may have a negative therapeutic effect. Fortunately, such situations are very rare [7,8,9,10,11,12,13,14,15,16]. It is yet to be known whether the commonly used criteria for the assessment of skin irradiation adequately correlate with the symptoms reported by the patient. They contain evaluation criteria that can be used by the Radiation Therapy Oncology Group (RTOG), Common Terminology Criteria for Adverse Events (CTCAEs), and World Health Organization (WHO) practitioners. They consider changes in skin biophysical parameters such as skin blood supply, pigmentation, hydration, pH, and symptoms reported by the patient, i.e., pain, itching, local heat, and pressure in patients with breast cancer undergoing radiotherapy. The examined patients are assessed according to the Common Terminology Criteria for Adverse Events [9]. This scale assesses the toxicity of treatment. It was created to try to standardize the reporting of adverse reactions in clinical trials and clinical practice. In the case of radiotherapy, it allows the assessment of the radiation reaction. Similarly assigned values are defined: 0: no changes; I: mild redness or dry exfoliation; II: moderate redness, limited wet exfoliation, moderate swelling; III: wet exfoliation in areas other than skin folds, bleeding after minor trauma; IV: necrosis and/or ulcerations with thinning of the skin, spontaneous bleeding; V: death. The same classification applies to patients who have undergone conserving surgery and those who have undergone mastectomy [11,12,13]. This method requires the experience of the attending physician. Additionally, it is impossible to observe the irradiation site in time and simultaneously document its change.

The energy dose accepted by tissue as well as other tissue, expected and non-expected skin reactions, usually results in temperature changes [10,11,12,13].

Currently, no methods available in clinical practice allow an objective assessment of the radiation reaction. Additionally, the problem is to define the area in which the irradiation dose is assessed and analyzed during radiotherapy. In the available literature, no studies evaluate thermography’s usefulness in assessing radiation reactions using thermography correlations between isodoses and the treatment area.

On the other hand, thermal imaging aims to obtain accurate thermal maps of the body. In medicine, it is successfully used as a non-invasive imaging of inflammatory diseases, breast cancer diagnostics [17,18,19,20,21,22,23,24,25,26,27], rheumatology, and various other applications, as well as to detect skin temperature changes [28,29,30,31,32,33]. Additionally, due to the fact that it is a non-invasive method, we can repeat it without harm to the patient. In addition, it provides us with additional information on whether the temperature changes during irradiation and in what areas. That is why thermal imaging seems to be a very convenient imaging technique to study temperature changes in the irradiated area, which indirectly brings information about energy absorbed or/and released in the tissue [34].

The main aim of the study was to compare the temperature response of the body to the dose received during breast cancer radiotherapy in patients after mastectomy and breast-conserving surgery. In addition, we want to check the correlation between isodoses read from treatment plans and isotherms obtained from the analysis of thermal images in two subgroups.

## 2. Materials and Methods

### 2.1. Consent of the Bioethics Committee

The work is part of the long-term project “Application of thermal imaging in cancer radiotherapy”, which was approved by the Bioethical Committee of the Oncology Center—Maria Skłodowska-Curie Institute (current name is the Maria Skłodowska-Curie National Research Institute (NIO-PIB) in Warsaw on October 6 2016 (No. 38/2016).

### 2.2. Control and Research Group

Two groups of patients were examined. The control group comprised 50 healthy patients (mean age 50 ± 12 years). The research group included 50 patients who were qualified for radiotherapy by the decision of a medical council. It consisted of two subgroups: 27 patients after conserving surgery (mean age 52 ± 11 years), and 23 patients after mastectomy (mean age 57 ± 13 years).

The power of the sample was checked for two research subgroups. For post-mastectomy patients the power of the test was 0.84. For a high power of 0.9, the number of patients in this group would have to be 29. For patients after partial surgery, the power of the test was 0.66. To obtain a high test power of 0.9, the number of patients in this group would have to be 50.

### 2.3. Measuring Device

All studies were performed with a thermal imaging camera FLIR System E60 model with a detector resolution of 320 × 240 pixels and a thermal sensitivity of 0.05 K. The conducted studies were free of charge, and volunteers could apply.

### 2.4. Patient Eligibility Criteria

Only healthy patients could qualify for the control group. The criteria for exclusion from the study were: consumption of alcohol, stimulants, and smoking for at least two hours before the study, intense exercise on the day of the study, infection with a body temperature above 37.5 °C, use of drugs that reduce body temperature, sunbathing on the day of the study, tests physiotherapy treatments performed, skin covered with ointments, creams, makeup or dirt, dermatological changes, and tattoos in the area covered by the examination [35,36,37].

### 2.5. The Method of Conducting the Test

The study was carried out at the Maria Skłodowska-Curie National Research Institute of Oncology Gliwice Branch in a special room designated for this purpose, which was not sunny and was closed for the duration of the examination to ensure the patient’s comfort. During each test, the staff monitored whether the temperature and humidity in the room remained constant, assuming the temperature values were 22 ± 1 °C and the humidity ranged from 40% to 45%.

Each person had to provide written consent to participate in the study. The staff provided detailed information about the survey and answered all questions. Each participant received a “Patient Information Form” describing all the study information. The last part before the examination was to complete a detailed patient questionnaire. The questionnaire of patients was extended to include the disease history (time of tumor detection, course of treatment, date of surgery). As thermography is non-invasive, it was decided that thermal imaging pictures would be taken each week of treatment. The meeting was always held in the same room, and the patients were reminded to prepare on the examination day. Each time they acclimatized to the ambient temperature twenty minutes before the test without upper garments. Meeting with the patients was held at a fixed time on a designated day of each week. The patients were always imaged before the administration of the fractional dose. Each week, the patients’ well-being was checked, the skin condition in the irradiated area was assessed, and possible side effects were noted. During thermal imaging, patients were always in the same position (standing with raised arms). According to standards, three straight projections were made from the left and right sides [35,36,37,38].

### 2.6. Analysis of Thermograms

In healthy patients who qualified for the study, the mean values of temperatures in the right and left breast were analyzed after the measurements were performed. It must be emphasized that the painted areas of the breast were adapted to the anatomy and structure of each patient.

**Figure 1:**
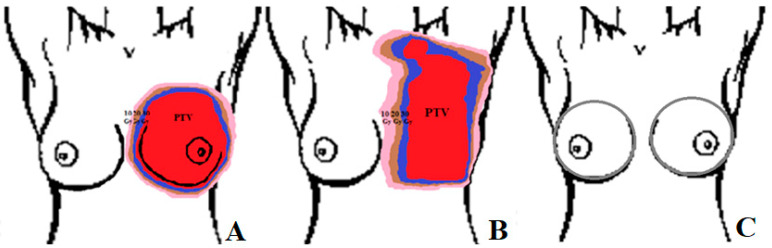
Diagram of the method of drawing isodoses in patients after conserving surgery (**A**) and mastectomy (**B**) qualified for radiotherapy. The PTV area is marked in red, the dose area of 30 Gy in dark blue, the dose of 20 Gy in brown, and 10 Gy is pink. Scheme of drawing the breast area on thermograms in a group of healthy patients (**C**).

In the research group, all patients underwent standard procedures to prepare for radiotherapy. A thermoplastic mask and computed tomography were made and calculated for the spatial distribution of the dose. The studied patients were treated five days a week with a fractional dose of 2.25 Gy, with a two-day weekend break for four weeks up to a total dose of 45 Gy. The area of PTV painted by the doctor is the area where the dose was provided. Then, isodoses were analyzed, i.e., lines connecting points with the same dose values. For the PTV area and the values of 30 Gy, 20 Gy, and 10 Gy, they were also marked on thermograms, and their changes were analyzed in each week of treatment. The diagram of the method of drawing isodose in patients treated with radiotherapy and the analysis of the temperature difference between the breasts in healthy women is shown in Figure 1.

The areas drawn by doctors differ depending on the surgical procedure performed. Patients, after conserving surgery, cover the area of the breast with a margin, and in the case of mastectomy, they also cover the lymph nodes. This confirms that the PTV area painting system proposed by us from the treatment plan is the most accurate. Especially in the case of mastectomy patients where determining the area after breast excision appears to be problematic and would not include areas outside the breast that also receive radiation doses. Therefore, individual preparation of isotherms in correlation with isodoses seems to guarantee repeatability and proper preparation of the analyzed area.

### 2.7. Statistical Analysis

Statistical analyses were performed using the STATISTICA 10 program, which contains a complete set of statistical tools and methods for comprehensive development and graphical presentation of the results of clinical trials. For each analysis, the Shapiro–Wilk test was performed to check the type of distribution of measurable features, and it was checked whether the distribution of the variables was normal and the homogeneity of variances. Based on those positive results, it was decided to perform parametric tests (including the student’s *t*-test for dependent groups in the case of, for example, the analysis of temperature changes over time or independently examining the differences in temperature between the groups of healthy and treated patients). The level of significance was *p* < 0.05. The confidence interval was 0.95. The results were presented using graph boxes. Pearson’s correlation was performed, which allows to determine whether two quantitative variables are related to each other by a linear relationship.

## 3. Results

**Figure 2:**
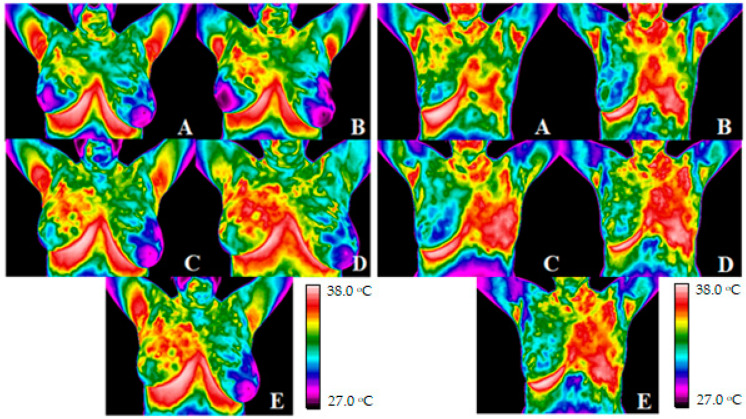
Thermograms of an exemplary patient after conserving surgery (**left**) and mastectomy (**right**) taken before radiotherapy (thermogram **A**), after the first week of treatment (thermal image **B**), after the second week of treatment (thermal image **C**), after the third week of treatment (thermal image **D**), and after the fourth week of treatment (thermal image **E**).

Pictures from Figure 2. present thermal images of an exemplary patient after conserving surgery and after mastectomy taken before radiotherapy (A) and in each subsequent week of treatment (B–E). The analyzed temperature range was set to 27–38 °C. One can see that the temperature in the irradiated area rises during radiotherapy. The highest temperature in the irradiated area was observed in both groups of patients in the third week of treatment.

Five measurements were performed for each patient in the research group. The target area was marked on the thermograms, PTV, then the isodoses of 30 Gy, 20 Gy, and 10 Gy. It can be easily seen that during the course of treatment, the temperature of the analyzed areas increases. The most significant irradiated tissue temperature increase is observed in the third week of treatment. As expected, a higher increase in mean temperatures occurs in the area where the highest dose is administered. One thermovision examination was performed for the control group, which consisted of healthy volunteers. For the analysis of the mean value of temperatures, the area of the right breast 33.14 ± 1.05 °C and the left breast 33.35 ± 0.99 °C were defined. The mean value of the differences between the breasts was 0.21 ± 0.05 °C and there was no statistically significant temperature difference between breasts.

**Figure 3:**
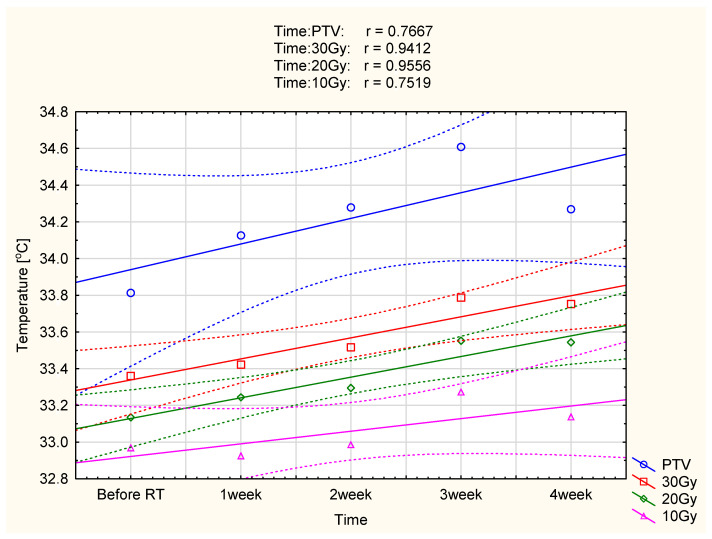
Correlation of temperature changes with the time of radiotherapy digestion for the PTV area and 30 Gy, 20 Gy, and 10 Gy isodoses in 23 patients after mastectomy. The confidence interval was 0.95.

**Figure 4:**
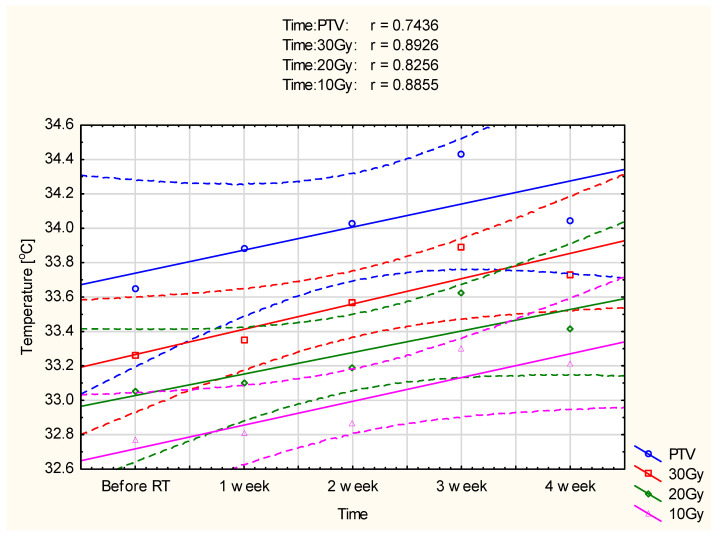
Correlation of temperature changes with the time of radiotherapy digestion for the PTV area and 30 Gy, 20 Gy, and 10 Gy isodoses in 27 patients after conserving surgery. The confidence interval was 0.95.

For deeper insight into performed analysis, the correlation between dose and mean adequate temperature areas was performed, and the plots are presented in Figure 3 and Figure 4.

It should be noticed that the lowest values of analyzed areas’ average temperature are observed before the start of radiotherapy. The mean temperature values increase after the first and second treatment weeks. However, the highest temperature was observed in the third treatment week. Additionally, in both groups there was an increase in temperature not only in the target area but also in the analyzed isodoses. The observed temperature increase was smaller for lower doses of energy delivered to the tissue. The obtained results showed a strong correlation between the mean temperature and the duration of radiotherapy in marked isodoses (so the dose of energy delivered) in patients after mastectomy and surgery, as shown in Figure 3 and Figure 4.

Table 1 Shows the temperature changes in the PTV and differences between the third week of treatment and the pre-radiotherapy temperature values for 20 exemplary patients, 10 exemplary patients in each of the studied groups.

**Figure 5:**
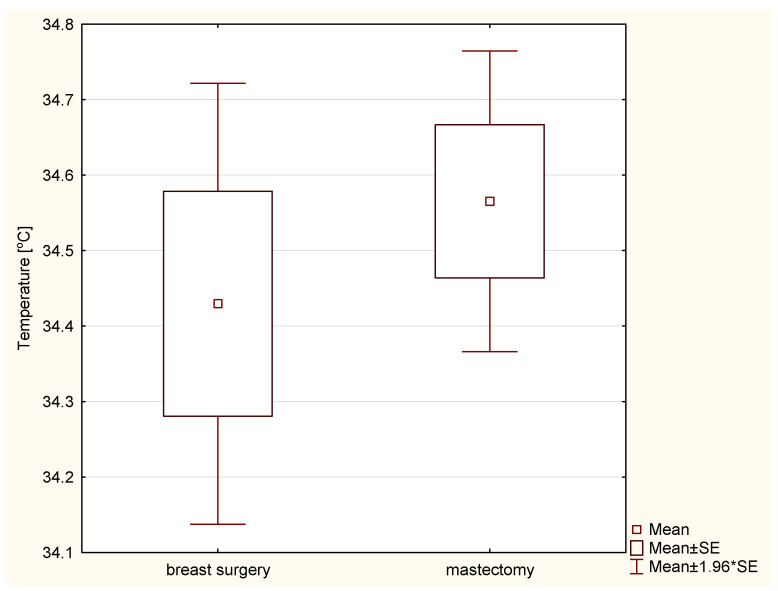
Average temperature of patients after breast conserving surgery and mastectomy in the PTV area after 3 weeks of radiotherapy.

Figure 5 shows the mean temperatures for the two groups in the third week of treatment in the treatment area (PTV). This week, both groups had the highest temperature measured during the weekly control of radiation therapy. There was no statistically significant temperature difference between the groups *p* = 0.471733.

**Figure 6:**
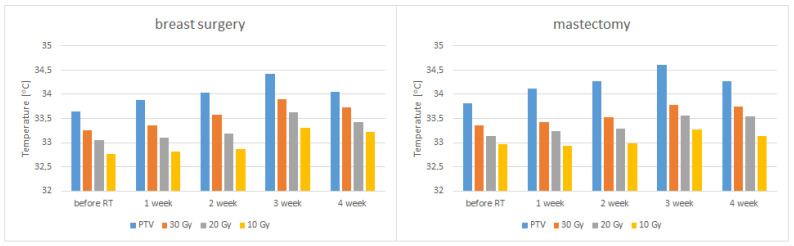
Mean temperatures of patients in the group after conserving surgery and after mastectomy in the area of PTV and isodoses.

Shown in Figure 6 bar graphs show the values of mean temperatures in the PTV area and isodoses before radiation therapy and in each treatment week. We can see that the average temperature values increase with each week, reaching the highest values in the third week of treatment. Temperatures increase not only for the target area that received the highest temperature, but also for the isodoses that received the lower cumulative dose. Comparing the two groups of patients, we can see that in the analyzed areas we have a similar increase in average temperature values. This confirms that the use of thermovision to monitor patients during radiotherapy treatment can be used both after mastectomy and partial surgery.

Compared with the pre-radiotherapy thermograms, the average temperature increase observed in patients after mastectomy was 0.8 ± 0.04 °C in the third week of treatment. The mean value for the examined patients before the start of radiotherapy was 33.81 ± 0.56 °C, and it increased up to 34.61 ± 0.43 °C. This statistically significant value *p* = 0.00369 confirms that the temperature increase observed during treatment results from ionizing radiation delivered to the tissue. With each week of treatment, the total dose delivered to the tissue is more remarkable, which is manifested by a higher temperature difference than before treatment. In the third week of treatment, when the recorded temperature was the highest in patients after conserving surgery, the average temperature increased by 0.78 ± 0.07 °C. The mean value before the start of treatment was 33.65 ± 1.03 °C, and it increased up to 34.43 ± 0.76 °C, these differences are statistically significant and amount to *p* = 0.000055. After removal of the tumor and the entire breast, the thermal response of the examined patients to the radiation dose was similar.

Additionally, the average temperature values obtained in the PTV area and other selected isodoses in the third week of treatment were compared. Such analysis showed similar dependencies in both groups. As the dose taken by the patient decreases, the increase in temperature is less. Statistically significant differences between the PTV values and each of the isodoses at the third week of treatment are presented in Table 2. Comparing these values between patients after conserving surgery and mastectomy, no statistically significant differences were found. However, it should be noted that the temperature rise in the low-dose areas is greater in patients after conserving surgery. This information confirms that in the analysis of thermograms after radiotherapy, it is worth not only determining the area painted by the doctor that receives the highest dose but also checking how the areas that received the lower dose of radiation behave. Additionally, in the course of patient monitoring through thermal imaging during the connection and noticing a significant increase in temperature in the area outside the PTV, it can indicate an incorrect implementation of the treatment which may result, for example, from the incorrect positioning of the patient on the therapeutic apparatus or appearing not expected of dose hot spots. We did not have such a case during the course of the study.

The mean temperatures between the PTV area in the third week of treatment, where the recorded increase was the greatest were compared, as was that of the untreated breast. In the group of patients after conserving surgery, this difference was 1.04 ± 0.19 °C and it is statistically significant *p* = 0.000008. This difference was five times higher than in healthy patients (where the temperature asymmetry between the left and right breasts was 0.21 ± 0.05 °C on average). In healthy women there was no thermal asymmetry between the studied areas, which is consistent with the literature [39,40]. The difference was even greater in the group of patients after mastectomy and amounted to 1.25 ± 0.14 °C. These differences are statistically significant, value *p* = 0.000005. Comparing these values between groups, we can see that they are not statistically significant. Such temperature results may suggest that modern radiotherapy spares the healthy side from treatment, so we do not observe an increase in temperature during treatment. In pre-radiotherapy patients, the difference between PTV treated area and healthy breast was 0.3 ± 0.05 °C for breast conserving surgery and was slightly higher in women after mastectomy was 0.4 ± 0.05 °C. This value is higher than in healthy women, but the differences are not statistically significant. Normal breast temperature did not increase in both groups during radiotherapy.

## 4. Discussion

The main goal of the work was to assess the usefulness of the infrared thermography method in assessing the body’s thermal reaction to the dose received during breast cancer radiotherapy. Since this treatment is performed after both conserving surgery and mastectomy, a necessary complement to the surgical procedure, it was decided to examine two groups of patients. To achieve this main goal, partial goals were set: correlating the areas delineated by isodoses from the treatment plan with the temperature image of the irradiated surface and observation of temperature changes in each week of treatment. After analyzing the results of our work, we obtained the following conclusions: In both groups of patients, there was an increase in temperature not only in the target area but also in the analyzed isodoses. With the decrease in the dose received, the temperature increase was smaller. Both in the case of mastectomy and after sparing surgery, painting the PTV area individually for each patient and weekly lesion analysis seems to be the most accurate method of assessing the patient’s response to the radiation dose. In the case of a mastectomy, it can be found that identifying the area to be analyzed can be confusing after the breast is removed. In this case, specifying the target area on thermograms allows for easier and unambiguous analysis. Until now, apart from an interview and visual assessment of the irradiated area by a physician, it has been difficult to obtain additional information on the patient’s response to radiation. The collocation of isodoses with isotherms allows us to quickly and non-invasively control the patient’s condition. It seems that in the event of an error in the treatment plan or improper setting during irradiation, the temperature change in the isodoses and PTV can show and help correct the error. There were no such patients in the study group.

All study patients were monitored for their health and well-being during treatment. In the study group, the most common symptoms were radiation reactions. In 58% of patients, it was classified according to the CTCAE scale [9,10,11,12,13] to value II and the remaining 42% to value I. An interesting observation was that the BMI value was lower among patients classified in group II (24.61) than in patients with a lower response to radiation (27.32). The second most common treatment-related side effect was fatigue and weakness, which occurred in 48% of the subjects. The above observations confirm that the ionizing radiation used during radiotherapy is not indifferent to the patient. Despite the technical difficulties and dose limitation on healthy organs, acute radiation dermatitis, called early skin reactions, occurs very often in clinical practice, may cause treatment discontinuation, and cause discomfort to the patient. Observation of side effects with the use of a non-invasive method, which is thermography, provides us additional opportunities. It lets the doctor know precisely when the reaction occurred and its change. In addition, these measurements can be performed during treatment, which allows for quick reactions and implementation of appropriate treatment. The differences in the values analyzed by us are not statistically significant between the groups, which indicates that the thermal reaction to radiation is the same both with conserving surgery and removal of the entire breast.

Our measurement method has several limitations. These include proper preparation of the patient, the measurement room, and the analysis of thermograms. We must be aware that the temperature is affected by many factors which is why, for example, an infection with increased body temperature disqualifies us from research during illness. We must take care and inform the patient about not using medications that may affect the change in body temperature. Correct performance of the tests requires having a specially prepared room, where the temperature and humidity of the air are constant, and should allow the patient to acclimate to the ambient temperature for about 30 min. Meeting these requirements is essential for reproducibility and reliable results

## 5. Conclusions

The studies confirmed the usefulness of the infrared thermography method to assess the patient’s response to the dose received in radiotherapy.

The proposed method of thermal maps according to the PTV area and isodoses allows for individual analysis of each patient and seems to be the most accurate. It is adapted to the patient’s anatomy and accurately reproduces the irradiated area. It seems to be particularly useful in the case of post-mastectomy patients, where determining the area for analysis may be problematic due to the lack of breasts.

An increase in temperature was noted in each of the analyzed areas during treatment, therefore it seems important to analyze not only the PTV area where the received dose was the highest, but also the isodoses where the received dose was lower. The highest temperature increase occurred in the third week of treatment in the PTV area. These data are consistent with literature values. The observed changes in temperature are similar both after mastectomy and after breast-conserving surgery.

A high positive correlation between isodoses and isotherms was obtained in the two analyzed groups.

## Figures and Tables

**Table 1:** Temperature changes in the PTV area shown in 20 exemplary patients. The calculated difference between the 3rd week of treatment and the mean temperature before radiotherapy and the differences between the breast temperature in the healthy group.

	Before RT [°C]	1 Week [°C]	2 Week [°C]	3 Week [°C]	4 Week [°C]	Differences between the 3rd Week of Treatment and before RT [°C]
mastectomy						
patient 1	33.5	34.0	34.2	34.4	33.9	0.9
patient 2	33.8	34.0	34.1	35.2	35.1	1.4
patient 3	33.3	33.5	34.1	34.2	33.8	0.9
patient 4	33.7	33.6	34.0	34.5	34.3	0.8
patient 5	33.3	34.1	34.3	34.6	34.4	1.3
patient 6	34.2	34.9	35.0	35.2	35.1	1.0
patient 7	33.6	33.7	34.0	34.5	34.4	0.9
patient 8	33.7	34.0	34.1	34.4	33.0	0.7
patient 9	33.6	33.8	33.9	34.2	33.8	0.6
patient 10	34.5	34.6	34.7	35.3	35.0	0.8
conserving surgery						
patient 1	33.0	33.4	34	34.9	34.5	1.9
patient 2	34.6	34.9	35.1	35.2	34.1	0.6
patient 3	31.7	31.8	31.9	32.4	31.7	0.7
patient 4	34.6	34.5	34.6	35.2	34.4	0.6
patient 5	34.4	34.7	34.7	35.0	35.0	0.6
patient 6	34.2	34.3	34.5	34.8	34.5	0.6
patient 7	32.7	33.8	33.8	34.0	33.8	1.3
patient 8	34.4	34.9	35.0	35.5	34.8	1.1
patient 9	34.2	34.0	34.1	34.8	34.1	0.6
patient 10	33.7	34.0	34.0	34.4	33.7	0.7

**Table 2:** Temperature increase for the PTV area, 30 Gy, 20 Gy, and 10 Gy in the third week of treatment in two study groups.

	PTV [°C]	30 Gy [°C]	20 Gy [°C]	10 Gy [°C]
mastectomy	0.80 ±0.04	0.43 ±0.09 *p* = 0.000798	0.42 ±0.08 0.000040	0.30 ±0.05 0.000000
conserving surgery	0.78 ±0.07	0.63 ±0.12 *p* = 0.040398	0.57 ±0.04 0.004271	0.53 ±0.11 0.000008

## Data Availability

Not applicable.
